# Self-assembled gel microneedle formed by MS deep eutectic solvent as a transdermal delivery system for hyperpigmentation treatment

**DOI:** 10.1016/j.mtbio.2024.101090

**Published:** 2024-05-14

**Authors:** Qi Zhao, Na Gu, Yier Li, Xia Wu, Qianqian Ouyang, Luming Deng, Hui Ma, Yuzhen Zhu, Fang Fang, Hua Ye, Kefeng Wu

**Affiliations:** a The Second Affiliated Hospital of Guangdong Medical University, Guangdong Medical University, Zhanjiang, 524003, China; b The Marine Biomedical Research Institute of Guangdong Zhanjiang, School of Ocean and Tropical Medicine, Guangdong Medical University, Zhanjiang, 524023, China; c Guangdong (Zhanjiang) Provincial Laboratory of Southern Marine Science and Engineering, Zhanjiang, 524023, China

**Keywords:** Hyperpigmentation, CUR, MS/DES gel, Self-assembly, CUR-MS/DES-GMN

## Abstract

Hyperpigmentation (HP) is an unfavorable skin disease that typically caused by injury, inflammation, or photoaging and leads to numerous physical and psychological issues in patients. Recently, development and application of natural whitening substances, particularly compound curcumin (CUR), is one of the most prevalent treatments for HP. However, it is still a formidable challenge to improve the percutaneous delivery of CUR due to its inadequate solubility in water and excellent barrier function of skin. To overcome the limitations of conventional delivery and increase the percutaneous absorption of CUR, the efficient delivery of CUR is urgently required. Herein, we developed a new malic acid-sorbitol deep eutectic solvent (MS/DES) gel microneedle loaded with CUR as a transdermal delivery system for HP treatment. The MS/DES gel produces three-dimensional (3D) network structure by self-assembly of hydrogen bond interactions, which conferred the CUR-MS/DES-GMN with sufficient mechanical properties to successfully penetrate skin tissue while also helping to enhance the drug's release rate. The CUR-MS/DES-GMN exhibit high biocompatibility and mechanical property in vivo of mice. The zebrafish experiments also show that CUR-MS/DES gel has significant effect of anti-pigmentation. Therefore, the designed CUR-MS/DES-GMN system provides a novel strategy for HP treatment based on self-assembly of naturally molecules.

## Graphical abstract

**Figure:**
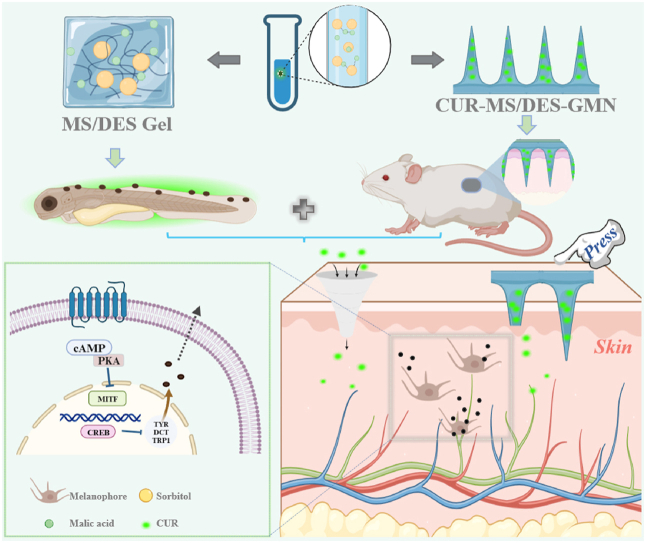

## 1 Introduction

Hyperpigmentation (HP) is a common cosmetic issue and dermatological condition characterized by an increase in skin color, frequently occurring following trauma, inflammation, burn injury, surgery and light aging [[1], [2], [3]]. Notably, skin diseases resulting from HP of exposed areas, such as melasma, freckles, and even melanoma, can significantly affect an individual's appearance. This can lead to substantial emotional distress, psychosocial burden, and potentially reduced quality of life for patients [4,5]. It is reported that tyrosinase (TYR) is the key rate-limiting enzyme during melanin synthesis, while tyrosinase-related protein 1 (TRP-1) and tyrosinase-related protein 2 (TRP-2) are involved in the catalytic process and play an important role in maintaining TYR homeostasis on melanosome membranes [6,7]. The microphthalmia associated transcription factor (MITF) is the most important transcription factor related to melanin synthesis, and its products are mainly involved in regulating the transcription of TYR, TRP-1 and TRP-2. The activation of MITF is dependent on the activation of cAMP-response element-binding protein (*p*-CREB), and *p*-CREB promotes the transcription of MITF, which in turn enhances the expression of TYR. Consequently, this leads to an enhancement in melanin synthesis [8,9]. Currently, the treatment methods for HP involve topical formulations of conventional drugs like hydroquinone [10], kojic acid [11], as well as glycolic acid [12] and oral formulations of therapeutic agents such as tranexamic acid [13], melatonin [14], and cysteamine hydrochloride [15]. However, there are certain limitations and obvious side effects such as skin erythema, peeling, drying or irritation. Curcumin (CUR, Fig. S1), a natural compound derived from plant polyphenols, has a wide range of biological activities, including anti-oxidation, anti-inflammatory, and antibacterial [16,17]. Furthermore, Jeon et al*.* found that CUR can reduce the *α*-MSH-induced melanin production and downregulate the expression of TYR, MITF, TRP-1, and TRP-2 genes associated with melanin production [16,18]. Regrettably, the transdermal drug delivery and oral bioavailability of CUR is limited by the superior barrier properties of the stratum corneum [19]. Hence, it is necessary to develop a drug delivery system to promote the transdermal penetration of CUR for the treatment of HP.

Microneedle patches (MNs) created using micron-scale needles in an array format and composed of various materials, has been studied as a novel transdermal drug delivery system (TDDS) [20,21]. MNs are normally utilized to penetrate the skin barrier and create micron-sized pathways (up to a depth of 100–200 μm) [22]. This process can guide and release targeted drugs (like CUR) into the dermis region, when self-dissolution, which can significantly improve the drug delivery efficiency. Besides, MNs would not penetrate deeper into the dermal layer, thereby avoiding damage to the blood vessels and nerves and minimizing pain sensation [23]. Therefore, MNs delivery system have been widely used in various fields because of its advantages such as minimally invasive, safe and high delivery efficiency [24,25].

Recent studies have demonstrated great potential for preparing MNs using a variety of materials, including metals, glass, and silicon [26]. These materials are rigid and can penetrate the skin with ease. In spite of these advantages, nevertheless, their brittleness poses a risk of breakage within the skin layer, which raises safety concerns [27]. For instance, silicon MNs-forming may break near the tip after inserting into skin [28]. Moreover, there is bad condition that leads to skin inflammation and secondary damage when minimal silicon-fragments with unproven biocompatibility remaining underneath the skin. The applicability of these types of MNs in clinical settings remains a topic for ongoing discussion. In recent times, the self-assembly of natural compounds (polymers or small molecules) to produce dissolving gel MNs is receiving increasing attentions in the field of pharmaceutical research [[29], [30], [31]]. Compared with MNs TDDS above, the DES gel MNs formed by natural compounds have attracted great attention in various fields due to its extensive and varying solubility profiles, adaptable polarity, inert chemical profiles, variable viscosities, biocompatibility, biodegradability and chemical stability [[30], [31], [32]]. These DES gel hardly induce harsh side effects [33]. Furthermore, DES, a novel system with proven capabilities promoted transdermal permeation of bioactive molecules (bovine serum albumin, ovalbumin, and insulin) without deleterious impacts on the cells [34,35]. Although the MNs composed of polymers such as sodium hyaluronate (HA), sodium carboxymethyl cellulose (CMC-Na), and glucans have been reported, a knotty problem is insufficient mechanical strength of most polymers [[36], [37], [38]]. Consequently, the task of designing self-assembled gel MNs TDDS with ideal mechanical strength and biocompatibility utilizing natural compounds remains a significant and formidable challenge.

**Scheme 1:**
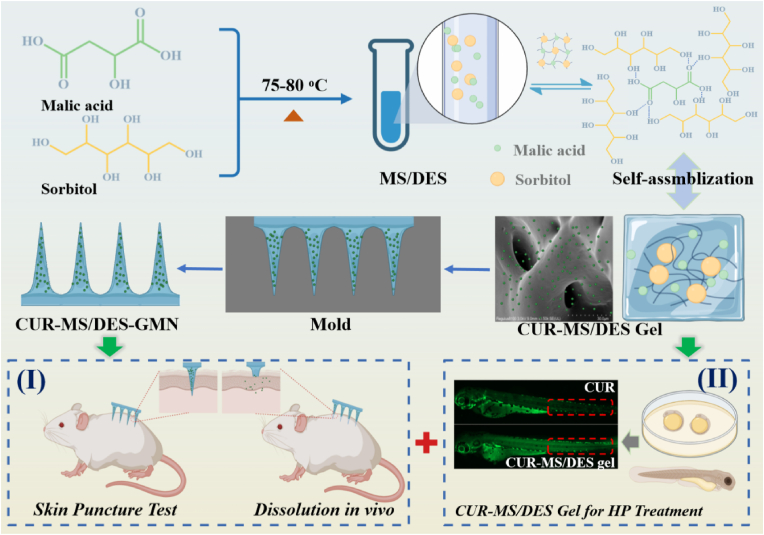
Schematic diagram of the formation process for CUR-MS/DES-GMN and its potential application as a TDDS for HP treatment.

In the present study, our group provides a new strategy to prepare a self-assembled MS/DES-GMN of malic acid and sorbitol via the hydrogen bonding interactions. To the best of our knowledge, it is the first report on MS/DES-GMN loading with CUR, which was used to improve the efficiency of the transdermal delivery of CUR and further applied for HP treatment by percutaneous administration. The CUR-MS/DES-GMN is constructed by a two-step casting and self-assembling of DES (MS) in specific molar ratios. The 3D hydrogen bonding network structure of MS/DES is able to adequate mechanical strength for CUR-MS/DES-GMN to easily penetrate skin tissue while also promoting effective release of CUR [39]. The results show that the CUR was evenly distributed in the needle part of the microneedle by light and confocal laser scanning microscopy, leading to the accuracy of the CUR delivery. To evaluate the mechanical property, biocompatibility and biodegradability of CUR-MS/DES-GMN, we conducted animal experiments using mice (Scheme 1). Moreover, zebrafish embryo tests were used to investigate the impact of the CUR-MS/DES gel on HP. The results revealed that the CUR-MS/DES gel significantly facilitating the transdermal delivery of CUR decreased the expression of cAMP response element binding protein (CREB), phospho-cAMP response element binding protein (P-CREB), microphthalmia-associated transcription factor (MITF) and tyrosine (TYR) proteins, which demonstrated that CUR-MS/DES-GMN is excellent to achieve the goal of treating HP. Hence, CUR-MS/DES-GMN is expected to provide a safe, alternative and promising method for HP treatment.

## 2 Experimental section

### 2.1 Materials

d-Sorbitol (purity ≥98 %), dl-malic acid (purity ≥99.5 %), polyvinylpyrrolidone (PVP, MW = 8000, with a K value of 16–18), CMC-Na, and CUR were obtained from Shanghai Macklin Biochemical Co., Ltd. Phosphate buffer saline (PBS, 0.01 M) was purchased from Biosharp for biological tissue cleaning. Leagene offers 4 % paraformaldehyde (4 % PFA) for live biological tissue fixation, and RIPA lysate was purchased from Beyotime. All other chemicals were of reagent grade unless stated otherwise. In addition, all chemicals were directly applied to the experiments as received without further purification.

### 2.2 Animals

All mice (male, Strain C57BL/6, weighing between 18 and 22 g) were purchased from Liaoning Changsheng Biotechnology Co., Ltd (SCXK (Liao) 2020-0001). Mice were housed in a temperature-controlled environment with a 12 h light/dark cycle at room temperature. The experimental zebrafish utilized were wild-type AB lineage specimens, raised in a laboratory zebrafish culture system with conditions referring to the Westerfield method. The male and female fish were separated at a ratio of 1:2 in the incubator, and they were placed before the light on the next day and started to spawn under the stimulation of light. Embryos were collected, rinsed with water, and selected for normal development under a SteREO microscope. They were then placed in a constant temperature incubator to proceed with the experiment. All animal experiments were approved by the Animal Experiment Ethics Committee of Guangdong Medical University (GDY2305001).

### 2.3 Preparation and characterization of MS/DES gel

By mixing sorbitol and malic acid in a molar ratio 4:1, and then heating under ultrasonic assistance at temperatures between 70 °C and 85 °C for a duration of 4–5 h, we were able to obtain a uniform and transparent MS/DES without any additional treatment. Subsequently, MS/DES was further placed in a drying oven for 2–3 days, during which the thermoreversible MS/DES gel formed via self-assembly for future use. The hydrogen bond interactions of MS/DES gel were demonstrated by nuclear magnetic resonance (^1^H NMR) Spectroscopy. Samples were prepared by dissolving 10 mg of malic acid and sorbitol or 20 mg of the freeze-dried MS/DES gel in 500 μL deuterated dimethyl sulfoxide (DMSO-*d*_6_), respectively. Then, the ^1^H and ^13^C NMR spectra were recorded on a Bruker AVANCE NEO 400 MHz solution-state NMR spectrometer (9.39 T). These spectra were subsequently processed using the MestReNova software. Moreover, Fourier transform infrared spectroscopy (FT-IR) spectra of malic acid, sorbitol and MS/DES were obtained using a FT-IR spectrometer (Bruker, Germany). The region studied covered the range of 4000–400 cm^−1^ with the resolution of 4.0 cm^−1^. And each spectrum was obtained by averaging 64 repeat scans. Besides, the surface microscopic morphology of MS/DES gel was observed by scanning electron microscopy (SEM, JSM-7610F, JEOL, Japan).

### 2.4 Rheological measurement of MS/DES gel

0.1 mg of CUR was incorporated into a uniform and clear 5.0 mL MS/DES. Subsequently, the CUR-MS/DES gel mixture was placed in a drying oven for a duration of 2–3 days. During this period, the CUR-MS/DES gel underwent self-assembly, forming a stable structure suitable for future applications. A rheometer was used to determine the glass transition temperature of MS/DES gel and CUR-MS/DES gel as it transitions from solid to liquid state. Liquid CUR-MS/DES is cured into CUR-MS/DES gel and its stability is demonstrated by rheological measurements. Temperature scan experiments were performed over a temperature range of 25 °C–90 °C at a rate of 4 ^o^C/min, a frequency of 1 Hz, and a strain of 1 % (in the linear range of the viscoelasticity of the material).

### 2.5 Differential scanning calorimetry (DSC)

DSC was carried out on sorbitol, malic acid, MS/DES and MS/DES gel to determine the phase transition processes, i.e. melting point and glass transition temperatures, from the heat absorption and exothermic peaks during heating and cooling. The DSC was operated in a Q2000 instrument (TA Instruments, USA) with a temperature range of 25–150 °C, a ramp rate of 10 ^o^C/min and a nitrogen flow rate of 10 mL/min.

### 2.6 Fabrication and characterization of CUR-MS/DES-GMN

First, 0.1 mg CUR was mixed into 5.0 mL of uniform and transparent MS/DES gel. To evaluate the stability of CUR-MS/DES gel, the distribution of CUR in MS/DES gel was observed by confocal laser scanning microscopy (CLSM) at 0, 7, 14, and 21 days. All samples were stored at 4 °C in a dark and dry environment. Next, this sample was filled into polydimethylsiloxane (PDMS) molds and centrifuged at 4000 rpm for 15 min in a high-speed cryo-centrifuge (Fresco 17, Thermo Scientific, USA). Then, the residual solution on the surface of the mold was removed and dried at room temperature (R.T.) for 2–3 days. PVP and CMC-Na were selected as the matrix materials for the MNs backing layer. An aqueous solution containing 10 % PVP and 2 % CMC-Na was prepared, and the PVP/CMC-Na solution was poured into the molds and centrifuged at 3500 rpm for 15 min. The CUR-MS/DES-GMN were peeled off from the mold after drying and stored in a desiccator until use. To observe morphology of the microneedles and the distribution of CUR, fluorescence images of microneedles in different xy-planes in the vertical direction (z-axis) were obtained using a confocal laser scanning microscope (IXplore spinSR, Olympus, Japan), and, subsequently, 3D reconstruction images were obtained by overlapping the xy-plane images along the z-axis.

### 2.7 Zebrafish embryo test

Zebrafish embryos (6–8 h after fertilization, hpf) were added to embryonic water containing CUR (5 μmol/L), MS/DES gel (0.25 %) and CUR (5 μmol/L)-MS/DES (0.25 %) at the shielding stage, and the embryonic water was changed and observed on a daily basis, and then the dead embryos were removed. After 72 h, live embryos were anaesthetized in a solution of tricaine methanesulfonate (0.02 %), and the embryos’ phenotypes were photographed at 72 hpf using a somatoscope (SZX7, Olympus, Japan). Following the recording process, zebrafish embryos (n = 40) were collected in centrifuge tubes and then placed at −80 °C for 20 min to sudden death. The zebrafish samples were lysed using radio immunoprecipitation assay (RIPA) lysis buffer (Beyotime, China), which was supplemented with a protease inhibitor and phosphatase inhibitor mixture (Beyotime, China). After centrifugation at 12,000g for 15 min at 4 °C, cellular protein was obtained. The concentration of this protein was determined using BCA protein assay kits (Biosharp, China). Cell lysates were then diluted in sodium dodecyl sulfate (SDS) sample buffer and the mixture was boiled for 5 min at 100 °C. Next, 20–30 μg of protein was separated by 10 % SDS polyacrylamide gel (Servicebio, China) electrophoresis and were then electroblotted onto a polyvinylidene difluoride (PVDF) membrane (0.22 and 0.45 μm, Millipore, USA). The blots were blocked with 5 % skim milk solution for 1 h at room temperature. Subsequently, these blots were incubated with primary antibodies against CERB, P-CERB, MITF, TYR and GAPDH. After incubation overnight at 4 °C, the blots were washed with tris-buffered saline (TBS) containing 0.1 % Tween-20. Thereafter, primary antibodies were detected through binding with HRP-conjugated secondary antibodies, and the proteins were visualized using a new super ECL kit (Beyotime, China) with the chemiluminescence apparatus, GV 1500 Pro II (BLT, China). The optical densities of the protein bands were semi-quantified with ImageJ software.

### 2.8 Zebrafish juvenile experiment

To investigate the ability of MS/DES gel to promote transdermal penetration of drugs, 5 dpf zebrafish were infiltrated in embryonic water containing CUR (5 μmol/L), MS/DES gel (0.25 %), and CUR (5 μmol/L)-MS/DES gel (0.25 %). The fluorescence content in zebrafish was observed using a confocal laser scanning microscope (IXplore spinSR, Olympus, Japan) and analyzed using Image-J.

### 2.9 In vitro insertion test

To observe the insertion ability, CUR-MS/DES-GMN was prepared and then inserted into the mice skin using a homemade applicator with a force of 10 N for 30 s. The insertion sites in the skin were observed under a confocal laser scanning microscope (IXplore spinSR, Olympus, Japan). The insertion rate (*I*_r_, %) was calculated according the following equation:

$$I_{r}=\frac{a}{A}\times 100\;\%$$

in which *I*_r_ is the insertion rate, a is defined as the number of pinhole sites on the skin after insertion and A is the number of needles in the array.

### 2.10 In vitro drug penetration studies

CUR-MS/DES-GMN were inserted into the mice skin using a homemade applicator with an applied force of approximately 10 N/patch and removed after 10 min. To visualize the drug penetration and the distribution of gel microneedles, with the help of fluorescent property of CUR (excitation/emission wavelengths of 425/530 nm), fluorescence images of different xy-planes in the vertical direction (z-axis) can be obtained from the skin surface using a confocal laser scanning microscope (IXplore spinSR, Olympus, Japan) with a gradual increase in height. Subsequently, 3D reconstructed images were obtained by overlapping the xy-plane images along the z-axis.

### 2.11 In vivo dissolution of CUR-MS/DES-GMN

Mice were anaesthetized to investigate the in vivo solubility of CUR-MS/DES-GMN. The CUR-MS/DES-GMN were then pressed into the back skin of the mice for a series of times. The height and dissolution trend of the microneedles were then observed by microscopy. The residual height (Y) of the microneedles at various time points (X) was graphed to represent a dissolution curve.

### 2.12 In vitro releasing experiment

Following the euthanasia of the mice and the removal of their hair using an electric razor, the dorsal skin was isolated. Subsequently, the adherent fat and other subcutaneous tissues were carefully excised. The prepared whole skin was then rinsed with saline and stored in a refrigerator at −20 °C with restricted use for one week. Prior to use, the skin was thawed by exposing it to the saline solution for 15 min and checked for skin integrity. In vitro skin permeation and retention experiments were performed using the Franz diffusion cell. The receptor chamber was filled with diffusion medium (pH 7.4 phosphate buffer and 40 % ethanol). The temperature of the diffusion cell was maintained at 37 ± 0.5 °C using a circulating water bath and the diffusion medium in the receptor chamber was continuously stirred with a magnetic bar at 300 rpm. The CUR solution, CUR+0.25 % MS/DES gel solution and CUR-MS/DES-GMN were gently placed on the donor side in an amount equivalent to 100 μg of CUR. At predetermined time intervals (0 min, 1 min, 2 min, 5 min, 10 min, 30 min, 60 min, 90 min, 120 min, 150 min, and 180 min), 1 mL of receptor fluid was collected. Subsequently, this volume was replaced with an equivalent solution to ensure a constant volume. The amount of CUR was measured using a UV–visible spectrophotometer with Ex = 442 nm and Em = 475 nm.

### 2.13 In vivo skin resealing and safety test

To study skin resealing and skin wound healing of CUR-MS/DES-GMN in vivo, the shaved mice was chosen to evaluate recovery test. CUR-MS/DES-GMN were pressed into the skin for 10 min and then removed and photographed. After euthanasia of the mice, the skin at the site of administration site was stripped off and fixed in 4 % PFA solution, then dehydrated, paraffin-embedded, sectioned, deparaffinized and stained with H&E. The sections were placed under the microscope to observe the healing of the internal skin tissues. Finally, the recovery of the deeper skin layers was observed under the microscope.

### 2.14 In vivo drug release

Mice skin was treated with CUR-MS/DES-GMN and then subjected to frozen sections. The skin was excised and washed with saline after administration for a certain time (0 min, 5 min, 10 min, 30 min, 60 min and 120 min). Slice samples of longitudinal sections of skin were prepared by cryosectioning (Leica CM 1950, Germany) and skin penetration of the preparation was observed using CLSM.

### 2.15 Animal treatments

The mice were acclimatized for 7 days prior to the experiment. Mice had their dorsal hair removed and exposed to ultraviolet radiation B (UVB) irradiation to induce HP (total energy dose per exposure = 1 J/cm^2^, wavelength = 306 nm, 3 times per week for 3 weeks). Then, the animals were randomly divided into five groups: control, model and MS/DES-GMN group, CUR solution group and CUR-MS/DES-GMN group. Next, mice in the MS/DES-GMN group and CUR-MS/DES-GMN group were given microneedle administration topically after UVB irradiation. Mice in the CUR solution group were treated with a coating of CUR solution. The skin condition of the mice was observed after three weeks of administration.

## 3 Results and discussion

### 3.1 Preparation and characterization of MS/DES gel

**Fig. 1:**
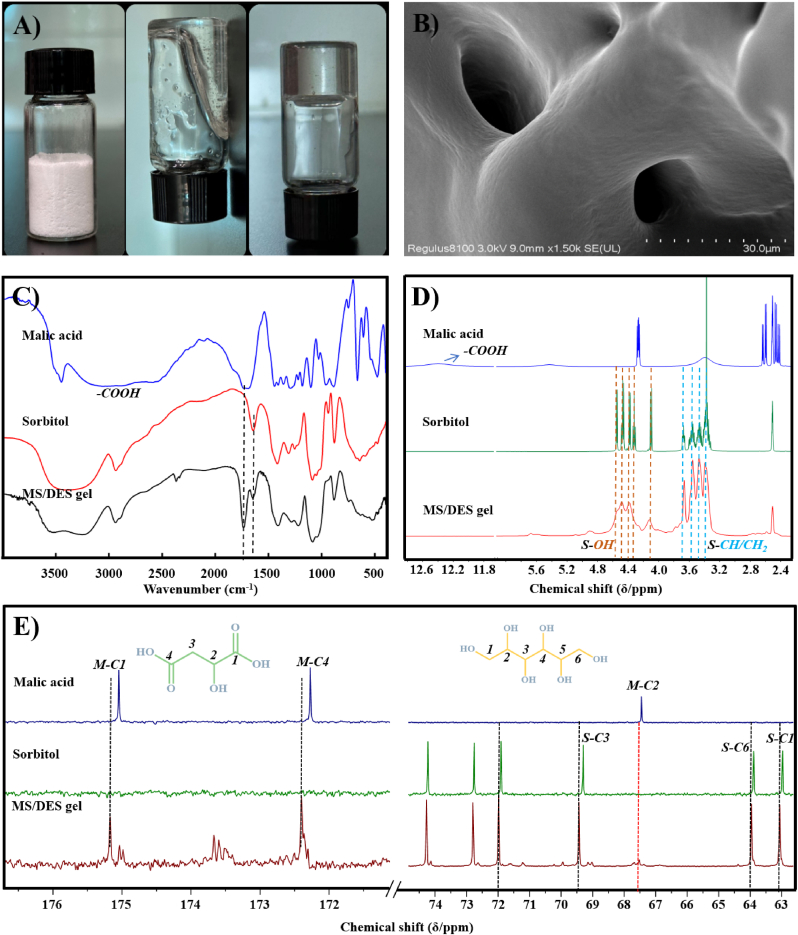
A) The photos of the mixture (malic acid and sorbitol, left), MS/DES (middle) and MS/DES gel (right). B) The SEM image of MS/DES gel. C) FT-IR spectra of malic acid, sorbitol and MS/DES gel. D) ^1^H NMR spectra of malic acid, sorbitol and MS/DES gel in DMSO-*d*_6_. E) ^13^C NMR spectra of malic acid, sorbitol and MS/DES gel in DMSO-*d*_6_. The letters M and S represent malic acid and sorbitol, respectively.

In this study, the components of the DES used herein, viz. malic acid and sorbitol, were selected due to their administration route and the intended efficacy improvement. Malic acid, extensively utilized in skin formulations, has the capacity to stimulate the metabolism of epidermal cells and expedite the removal of early fine lines on the skin [40]. Sorbitol is a hypertonic solution that has a moisturizing effect and allows the skin to retain moisture, helping to relieve dry skin [41]. Malic acid as a natural organic acid was used as the H-donor, while sorbitol was used as the H-acceptor to prepare the DES. The molar ratio of the H-acceptor/H-donor was set to 4:1. This stable and transparent MS/DES was prepared by the heat-method and CUR was further added to the MS/DES solution. Then, the mixture of CUR and MS/DES is placed in a drying oven for 2–3 days, and the originally liquid MS/DES self-assembles into MS/DES gel (Fig. 1A). The MS/DES gel was characterized in terms of its morphology and mechanical properties.

FT-IR spectrum results of MS/DES gel were shown in Fig. 1C. It is specifically to verify that, in the spectrum of sorbitol, there is an absorption band at 3486–3279 cm^−1^, which corresponds to the hydroxyl groups (O–H) stretching vibration and the peaks corresponding to the C–H stretching in the region of 2980–2812 cm^−1^. For malic acid, the O–H stretching vibration absorption of free carboxylic acid is located at 3500 cm^−1^, and due to the formation of dimers, the carboxylic peak is red shifted, forming a wide and scattered peak at 3200–2500 cm^−1^. The C=O stretching vibration of the dimer carboxylic acid is located at 1710^−1^. In addition, the peak at 920 cm^−1^ belongs to the OH⋯O, a relatively strong broad peak, which is the non-plane rocking vibration of the O–H of the two molecular association indicating that malic acid tends to exist in morphology of dimmers. By comparing the FT-IR spectra of the pure compounds with that of the MS/DES, it is possible to observe that the MS/DES spectrum is more similar to that of sorbitol. In the MS/DES gel spectrum, we found that the establishment of hydrogen bond interaction between malic acid and sorbitol causes a blue shift of –COOH group from 3200 to 2500 cm^−1^ to 3640-3180 cm^−1^ and the vibration of C=O group from 1710 cm^−1^ to 1760 cm^−1^, which indicated the dimer carboxylic group was broken.

To further reveal the formation of hydrogen bond between malic acid and sorbitol, the ^1^H NMR and ^13^C NMR results of malic acid, sorbitol and MS/DES gel were recorded (shown in Fig. 1D and E). Compared to malic acid and sorbitol, the ^1^H NMR spectrum of MS/DES gel shows that both components maintain their structures after the MS/DES preparation. However, it is clear to observe that the typical group –COOH at 12.41 ppm of malic acid and the multiplet structure of –OH region (3.90–4.78 ppm) from sorbitol were disappeared in Fig. 1D. Besides, the signals of –CH– and –CH_2_– between 3.10 and 3.88 ppm could not distinguish the fine structure, indicating that the hydrogen bond was formed. In order to determine the structure changes of MS/DES components, ^13^C NMR (Fig. 1E) was used to characterize MS/DES gel sample. It can be seen that the ^13^C signals at 175.17 ppm, 172.40 ppm, 69.44 ppm, 63.96 ppm, and 63.06 ppm have significant changes with the corresponding values of 0.13 ppm, 0.13 ppm, 0.14 ppm, 0.07 ppm and 0.09 ppm, which were assigned to M-C1, M-C4, S–C3, S–C6 and S–C1, respectively. Therefore, the NMR results demonstrated that the hydrogen bond interactions mainly occur between –COOH of malic acid and –OH (C1, C3 and C6) of sorbitol.

The micro-morphology of the MS/DES gel system was also evaluated. It was found that the MS/DES gel showed a dense and homogeneous structure, indicating that its cross-linking density was increased (Fig. 1B). This might be related to the formation of the MS/DES, which fills the voids in the gel and makes the connection tighter due to the generation of hydrogen bonds, which enhances the cross-linking inside the gel.

**Fig. 2:**
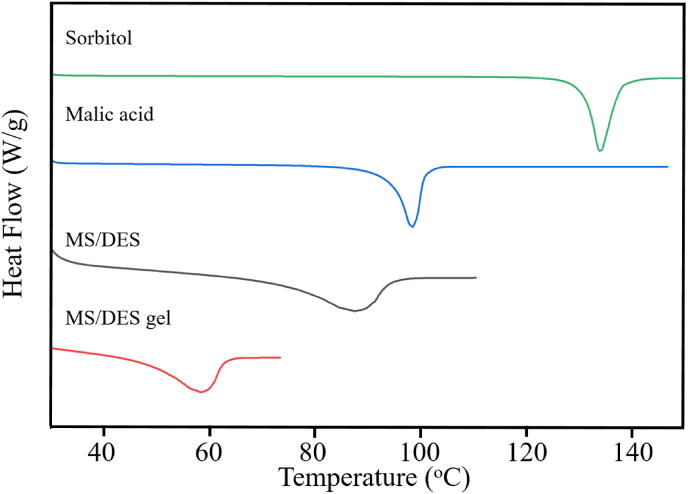
DSC plots of malic acid, sorbitol, MS/DES and MS/DES gel.

The MS/DES gel was further characterized by differential scanning calorimetry (DSC, Fig. 2). The malic acid, sorbitol and the fixed proportion mixture (MS/DES) demonstrated endothermal peaks at 134 °C, 98 °C, and 85 °C, respectively. However, there is a small endothermal peak at 58 °C in the thermogram of MS/DES gel, indicating the melting point of MS/DES gel, which is apparently lower than that of either malic acid or sorbitol.

**Fig. 3:**
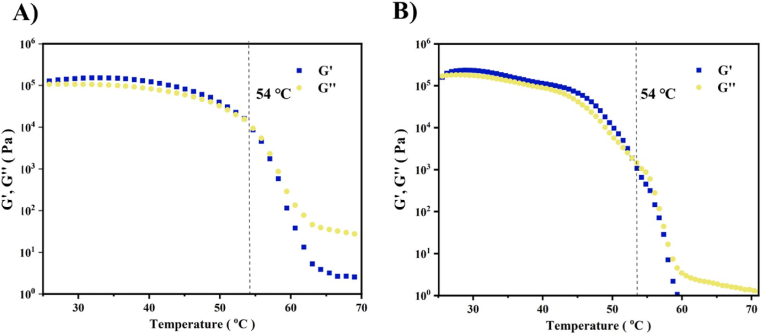
A) Rheological characterization of MS/DES gel. B) The rheological characterization of CUR-MS/DES gel. The rheological experiments were performed at a frequency of 1 Hz and a strain of 1 % (within the linear range of the material's viscoelasticity).

The MS/DES gel storage modulus (G′) and loss modulus (G″) varies with temperature was measured (Fig. 3). According to the results, as the temperature increases, the G′ always higher than G″ until 54 °C, which indicates that the increase of temperature does not destroy the bulk phase structure and it is more inclined to elastic solid. As the temperature continues to increase, G′ declines more rapidly than G″ and G′ remains lower than G″, which means that the internal structure of the colloid collapses locally or as a whole, gradually transforming from a viscoelastic solid to a liquid. Gel to sol transition temperature T_gel-sol_ is 54 °C indicates the applicability of the MS/DES gel at room temperature. In order to better elucidate its stability and reliability, the rheological characterization of the CUR-MS/DES gel was conducted (Fig. 3B and Fig. S2), showing that T_gel-sol_ remains unchanged despite the loading of CUR by MS/DES gel. In addition, it was observed that the absolute values of both moduli are relatively high (the average value of the storage modulus in the linear region was about 39,000 Pa), indicating the relatively high mechanical strength of the MS/DES gel and CUR-MS/DES gel [[42], [43], [44]].

### 3.2 In vivo assessment of transdermal permeation capability and anti-pigmentation in zebrafish of CUR-MS/DES gel

**Fig. 4:**
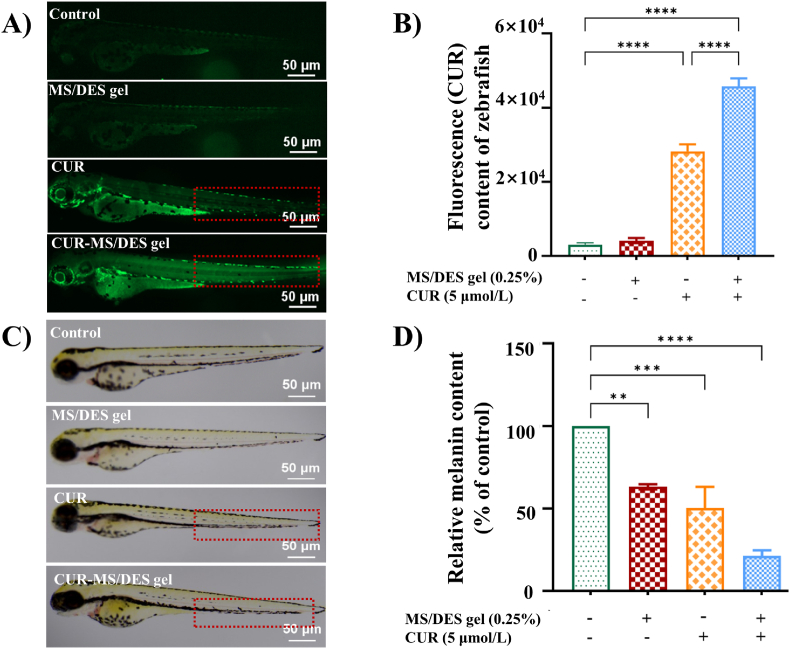
The study of the capacity of MS/DES gel to promote transdermal penetration of CUR. A) and B) Evaluation of drug penetration ability of MS/DES gel for CUR, **p < 0.01, ***p < 0.001, ****p < 0.0001 as compared to the control group. C) and D) Effect of CUR-MS/DES gel on melanin content of zebrafish embryos, **p < 0.01, and ***p < 0.001, ****p < 0.0001 as compared to the control group.

The skin permeability of the CUR-MS/DES gel system was evaluated in zebrafish using CUR as a fluorescent probe. Clearly, no signal can be found under the treatment of aqueous and MS/DES solution (Fig. 4A). When CUR was dissolved in MS/DES gel, they showed remarkable penetration enhancement with a broad and strong distribution of CUR after skin administration 15 min. Also the fluorescent signal of CUR in the MS/DES gel system was significantly enhanced compared with that in CUR solution. In particular, MS/DES can act as both a solvent and penetration enhancer. It has been previously suggested that solvent permeating through the skin could “drag” the permeant along with it, and the interaction between the solvent and the solute is proved to be the key influencing factor of the “drag” effect [45]. In the CUR-MS/DES gel system, CUR participated in the formation of MS/DES, a large number of hydrogen bonds were generated amount the CUR, H-receptors and H-donors, leading to a more stable system. Thus, the strong interactions in the CUR-MS/DES gel system resulted in an improved “drag” effect and skin-permeation enhancement. Taken together, the penetration-enhancing effect of MS/DES on the CUR might be attributed to the following factors. Firstly, the MS/DES system showed a stronger dissolution ability of the CUR compared to traditional solvents. Furthermore, the MS/DES exhibited high skin permeability and a moderate impact on skin barrier function. Finally, MS/DES afforded a strong interaction with the CUR, which could “drag” the CUR to penetrate through the skin at the same time, thus exerting a penetration enhancing effect.

Considering the prolonged duration and intricate procedures associated with mice experiments, we therefore chose zebra fish as an animal model to evaluate its pigmentation reduction capacity of CUR-MS/DES gel. Zebrafish embryos were anaesthetized after continuous administration for 72 h. Ten zebrafish were selected randomly for each group to observe the back discoloration, and then we used Image J software to evaluate the melanin on the backs of four groups of zebrafish quantitatively. The results of Fig. 4C and D shows that the pigmentation on the backs of treated zebra fish were lighter than those in the control group, the de-coloration rate in the CUR group is 50 %, while the de-coloration rate in the CUR-MS/DES gel group up to 70 %. Therefore, the CUR-MS/DES gel plays a very important role in the process of CUR inhibiting melanin production.

**Fig. 5:**
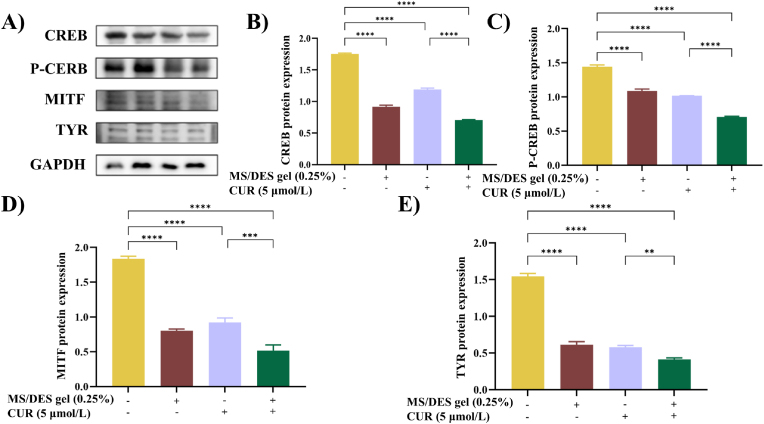
CUR-MS/DES gel inhibits the levels of proteins associated with melanin production in zebrafish. A) Levels of B) CREB, C) P-CREB, D) MITF, and E) TYR proteins in zebrafish treated with CUR-MS/DES gel using western blotting. Values are expressed as the means ± SD (n = 3). **p < 0.01, ***p < 0.001, ****p < 0.0001 as compared to the control or the CUR-treated group.

In order to explore the effect of CUR-MS/DES gel on the protein expression levels of pigmentation differentiation, we performed western blot to examine the expression of key regulatory factors. As shown in Fig. 5, CUR was found to inhibit the expression of CREB, P-CREB, MITF and TYR, especially the CUR-MS/DES gel group exhibited a stronger inhibitory trend due to the MS/DES enhanced transdermal delivery. The expression levels of CREB, P-CREB, MITF, and TYR exhibited a significant decrease in the groups treated with 0.25 % CUR (5 μmol/L)-MS/DES gel. Their enhancement compared to the control group was statistically significant. These qualitative and quantitative results of the zebrafish experiment preliminarily verified that CUR has de-coloration potential and MS/DES gel system further enhanced the permeability of CUR.

### 3.3 Stability of CUR-MS/DES gel

**Fig. 6:**
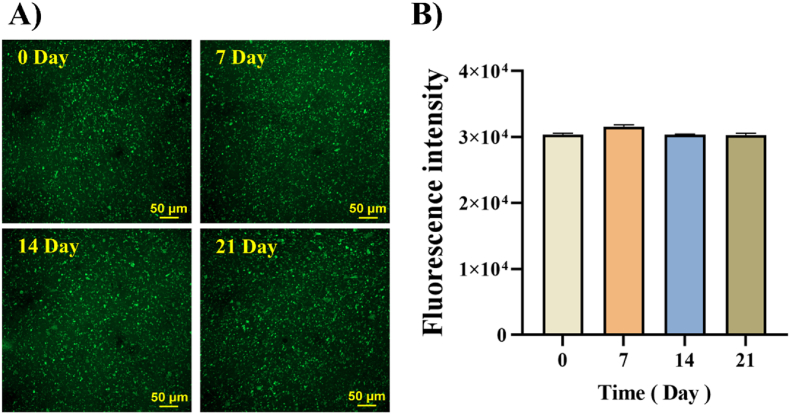
A) CLSM images of the distribution and B) fluorescence intensity of CUR in MS/DES gels at 0, 7, 14, and 21 days.

To assess the stability of the CUR-MS/DES gel, the distribution of CUR in the MS/DES gel was observed at different times (0, 7, 14 and 21 days). It was found that the fluorescence intensity of CUR in the MS/DES gel is not quenched or even significantly reduced with time (Fig. 6). The distribution results of CUR in the MS/DES gel demonstrated stability and homogeneity.

### 3.4 Preparation and properties of CUR-MS/DES-GMN

**Fig. 7:**
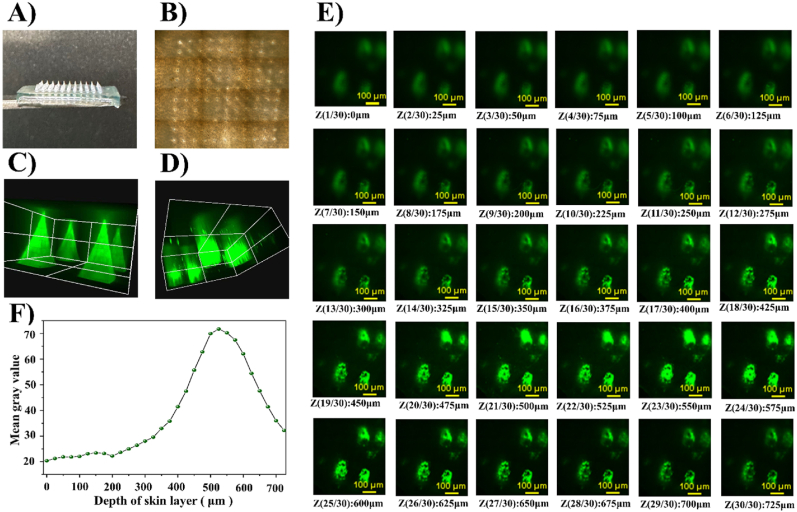
A) Morphology of the CUR-MS/DES-GMN. B) Microneedle puncture image of the puncture through the mice skin. C) 3D image of CUR-MS/DES-GMN. D) 3D reconstructed images of in vitro drug penetration of CUR-MS/DES-GMN. E) CUR distribution in different depths of the mice skin. F) Analysis diagram of fluorescence different levels.

To further improve the transdermal delivery efficiency, CUR-MS/DES-GMN arrays (Fig. 7A) were directly created by utilizing a straightforward two-step casting technique. Fig. S3 shows the fabrication procedure, master templates are first prepared by micro-fabrication methods on PDMS (micropore number: 11 × 11, high: ∼800 μm, length: ∼410 μm). Effective skin penetration requires a sufficient amount of mechanical strength. The insertion performance of the CUR-MS/DES-GMN was assessed using full thickness mice skin. The mice skin was spread on the filter paper with the cuticle 10 N facing outwards, the CUR-MS/DES-GMN was pressed vertically with a certain force for 30 s, and the CUR-MS/DES-GMN was removed. It was easy to distinguish whether the holes were left by the penetration of CUR-MS/DES-GMN in Fig. 7B. The holes formed by CUR-MS/DES-GMN in the mice skin were clearly visible. Then the holes left on the surface of the skin were counted, and the puncture rate was calculated to be about 90.5 % (±2.5), indicating that the CUR-MS/DES-GMN have sufficient mechanical strength to penetrate the stratum corneum of murine skin.

The capabilities of the obtained CUR-MS/DES-GMN to penetrate skin and CUR diffusion are investigated using full thickness mice skin. The fluorescence microscopy image before and after CUR-MS/DES-GMN insertion for 10 min has been provided in Fig. 7C and D. As Fig. 7C depicted that the green fluorescence of the CUR-MS/DES-GMN can be clearly observed due to the CUR has been encapsulated into the CUR-MS/DES-GMN. After CUR-MS/DES-GMN insertion, the green fluorescence at the surrounding area of encapsulated also can be observed, indicating rapid permeation and diffusion of CUR from the CUR-MS/DES-GMN in vivo. Also the green distribution of CUR is primarily at the needle part, while the basal portion is nearly nonfluorescent. The results indicated that drugs can be delivered to the skin more efficiently, which may reduce drug wastage. To visualize the penetration depth of CUR-MS/DES-GMN into mice skins and survey the diffusion of CUR at different depth regions, the skin tissues are further observed using CLSM, as shown in Fig. 7E. Images are collected from xy-plane with a step size in z axis of 25 μm from the stratum corneum to dermis layer until dark in visual at 725 μm. As shown in Fig. 7F, the fastest drug dissolution and the most robust distribution were observed at the skin layer of 525 μm.

### 3.5 In vivo drug delivery and dissolution

**Fig. 8:**
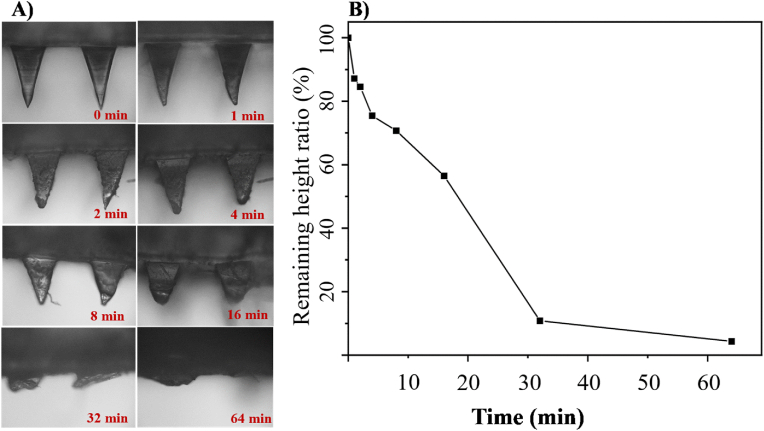
A) SteREO microscope images of CUR-MS/DES-GMN and B) Remaining height ratio (%) of needle after insertion into mice skin at different time periods.

The rate of CUR release is influenced by the degradation rate of CUR-MS/DES-GMN. The degradation of CUR-MS/DES-GMN was monitored using a SteREO microscope, as shown in Fig. 8A, and the degradation rate represented by the ratio of CUR-MS/DES-GMN remaining height in shown in Fig. 8B. After the CUR-MS/DES-GMN were inserted into the skin, MS/DES-GMN was degraded into natural small molecules (malic acid and sorbitol). As the insertion time increases, the CUR-MS/DES-GMN dissolve and shorten in length as expected. In addition, the height of all needles remains almost equal at the same time, indicating that all needles have a uniform structure and dissolve at the same rate in the skin. Within a mere 2 min, the needle tips underwent significant deformation and blunting. After 16 min of insertion, approximately 50 % of the needle had degraded. By the time it reached 64 min, the remaining height ratio of needle is 4.34 %, which demonstrates that needle had almost completely degraded. Consequently, the quick release of CUR was observed in tandem with the degradation of CUR-MS/DES-GMN.

### 3.6 In vitro releasing experiments

**Fig. 9:**
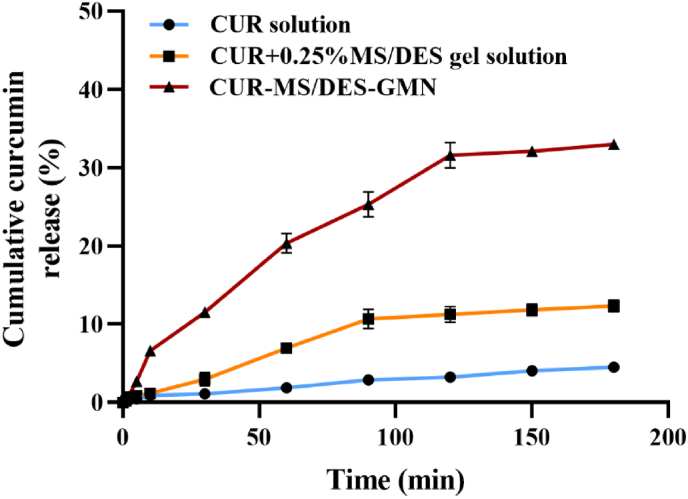
Cumulative release percentage of the CUR from CUR+0.25 % MS/DES gel solution, CUR-MS/DES-GMN, and CUR solution through the mice skin at different time intervals using a Franz diffusion cell. Results were represented as mean values ± standard deviation (mean ± SD, n = 3).

The main objective of this study was to promote skin penetration and deposition of CUR. In vitro drug penetration of CUR solution, CUR+0.25 % MS/DES gel solution and CUR-MS/DES-GMN was performed through the dorsal skin of mice. Fig. 9 shows the cumulative amount of CUR infiltrated into excised mouse skin over time. When CUR solution was placed directly on top of the skin as a control, CUR was first detected across the skin after 30 min, and its cumulative percentage release was only 4.5 % (±0.1 %) after 180 min. The CUR+0.25 % MS/DES gel solution penetrated the skin better than CUR solution, and CUR was first detected across the skin at 10 min. In contrast, CUR from the CUR-MS/DES-GMN the cumulative percentage release was 32.98 % (±0.62 %) after 180 min. The ability of CUR-MS/DES-GMN to penetrate the skin was stronger than that of the CUR solution, suggesting that CUR-MS/DES-GMN can achieve higher drug concentrations in the skin, and is ideal for the effective treatment of localized diseases.

### 3.7 In vivo drug release

**Fig. 10:**
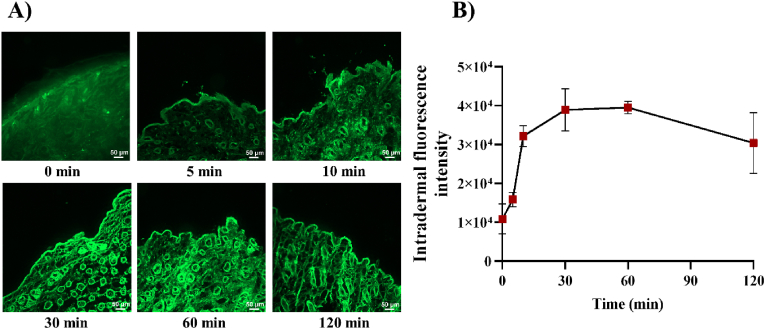
A) The images obtained from CLSM of longitudinal sections of mice skin after fixation using CUR-MS/DES-GMN. B) Fluorescence intensity analysis graph. Results were represented as mean values ± standard deviation (mean ± SD, n = 3).

In vivo skin penetration studies were performed utilizing CLSM images to investigate the effects of CUR-MS/DES-GMN on skin penetration, retention and intradermal drug distribution. As shown in Fig. 10, CLSM images of longitudinal sections of mice skin showed that faint fluorescence was observed in the skin and hair follicles 5 min after microneedle insertion. The intensity of intradermal fluorescence increased with increasing insertion time. Many intense fluorescent spots appeared in the skin and hair follicles after 1 h of insertion. After 2 h, there was a relative decrease in fluorescence intensity. Consequently, the results obtained from fluorescence microscopy were consistent with those observed during in vitro skin penetration, indicating that CUR-MS/DES-GMN was effective in facilitating both drug penetration and retention within the skin.

### 3.8 In vivo skin puncture test and safety test

**Fig. 11:**
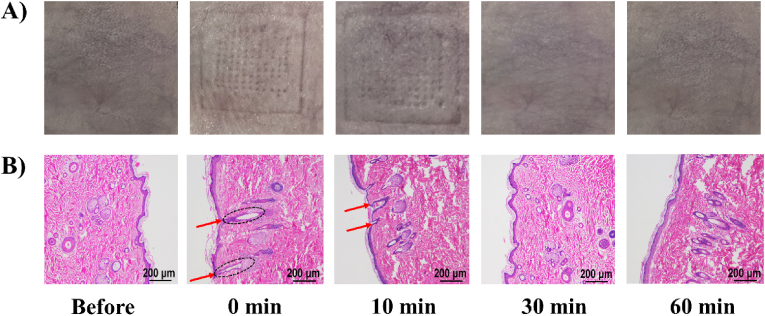
H&E staining of inserted skin sections A) before and B) after CUR-MS/DES-GMN.

The skin recovery performance after microneedle use is an important index for studying the safety of microneedle administration [46]. The CUR-MS/DES-GMN was inserted into the back skin of the mice, as time went on, the holes formed by CUR-MS/DES-GMN on the skin of living mice gradually healed and disappeared. H&E staining was performed on the puncture site to further evaluate the penetration of the CUR-MS/DES-GMN into the skin. The H&E staining results are shown in Fig. 11, which confirmed that the stratum corneum is broken after the CUR-MS/DES-GMN inserted the skin. Meanwhile, we also observed the continuous changes of CUR-MS/DES-GMN under the mice skin and the degree of wound healing (Fig. S4). The tip of CUR-MS/DES-GMN can penetrate the granular layer and delve deeper into the dermis layer of the skin. Notably, no adverse reactions such as redness, swelling, or inflammation were observed on the surface of mice skin during the skin puncture test. When the microneedles were removed, there were obvious pinholes on the surface of the skin, which gradually disappeared after 10 min, and the skin recovered after 30 min (Fig. 11A). In order to observe the recovery of the skin, H&E staining was also performed on the skin. Skin tissue sections at 0 min showed that the micropores formed by the microneedles could be clearly observed. After 10 min, the micropores formed by the microneedles were gradually reduced, and the skin was basically completely recovered after about 1 h (see Fig. 11B). Moreover, there was basically no trace of skin damage on the skin surface of the mice after 60 min of CUR-MS/DES-GMN insertion. The results (Fig. S4B) obtained from H&E analysis of CUR-MS/DES-GMN inserted into mice skin for 24 h also indicated that the subcutaneous micropores were completely absent. Hence, the skin puncture experiment and skin wound healing strongly demonstrated that the CUR-MS/DES-GMN exhibit high biocompatibility, biodegradability and safety in vivo of mice, which has great potential for the treatment of HP.

### 3.9 In vivo therapeutic effect of CUR-MS/DES-GMN for HP treatment

**Fig. 12:**
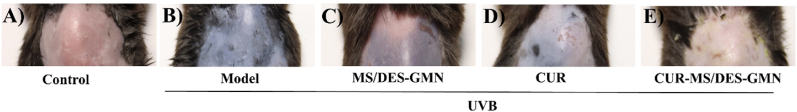
Pictures of CUR-MS/DES-GMN before and after for HP treatment in vivo.

Finally, the therapeutic effect of CUR-MS/DES-GMN for HP treatment in vivo by penetrating dorsal skin with pigmentation was further evaluated (Fig. 12). The results show that the skin chroma has been mildly restored in the MS/DES-GMN group. In comparison to the CUR solution (Fig. 12D), the chroma of the CUR-MS/DES-GMN group (Fig. 12E) has been significantly restored and is closer to the original color (Fig. 12A). Therefore, the results of in-vivo experiment demonstrated that CUR-MS/DES-GMN can effectively promote the transdermal penetration of CUR and its anti-pigmentation therapeutic effect.

## 4 Conclusion

In this work, we first report the direct self-assembly of MS into MS/DES gel, which can be further fabricated into CUR-MS/DES-GMN as a TDDS for HP treatment. The 3D network structure of MS/DES gel produced by noncovalent interactions on carboxyl groups of malic acid and hydroxyl of sorbitol, endows CUR-MS/DES-GMN with sufficient mechanical properties to successfully penetrate skin tissue while also enhancing delivery rate of CUR. The results of zebrafish experiments and mice skin therapeutic effect indicated that the CUR-MS/DES gel/CUR-MS/DES-GMN can significantly enhance the transdermal delivery of CUR. Meanwhile, Western blots experiments show that the combination of CUR and MS/DES gel is powerful to inhabit the protein expressions of CREB, P-CREB, MITF and TYR. It is important to highlight that the CUR-MS/DES-GMN not only possesses the benefits of self-assembly and high mechanical strength, but also demonstrates superior biocompatibility. This is evidenced by its in vivo skin puncture test and skin wound healing capabilities, further attesting to its biodegradability and safety. In conclusion, this study provides a novel approach to the design of TDDS (MS/DES-GMN) based on the self-assembly of natural molecules with CUR loaded, which significantly enhances transdermal efficiency and broadens their applicability in various biomedical applications related to HP treatment.

## CRediT authorship contribution statement

**Qi Zhao:** Writing – review & editing, Writing – original draft, Methodology, Investigation, Formal analysis, Data curation, Conceptualization. **Na Gu:** Methodology, Investigation, Formal analysis. **Yier Li:** Software. **Xia Wu:** Software. **Qianqian Ouyang:** Methodology. **Luming Deng:** Data curation. **Hui Ma:** Formal analysis. **Yuzhen Zhu:** Software. **Fang Fang:** Methodology, Software. **Hua Ye:** Supervision, Conceptualization. **Kefeng Wu:** Writing – review & editing, Supervision, Project administration, Methodology, Investigation, Funding acquisition.

## Declaration of competing interest

The authors declare that they have no known competing financial interests or personal relationships that could have appeared to influence the work reported in this paper.

## Data Availability

No data was used for the research described in the article.
