# Supplementary material for: Self-assembled gel microneedle formed by MS deep eutectic solvent as a transdermal delivery system for hyperpigmentation treatment
Source: Mater Today Bio. 2024 May 14;26:101090. doi: 10.1016/j.mtbio.2024.101090 (PMC11127278; doi:10.1016/j.mtbio.2024.101090)
Supplement: Multimedia component 1 [file mmc1.docx]

Supplementary Material

Self-Assembled Gel Microneedle Formed by MS Deep Eutectic Solvent as a Transdermal Delivery System for Hyperpigmentation Treatment

Qi Zhao ^a,b,c1*^, Na Gu ^a,b,c1^, Yier Li ^a,b,c^, Xia Wu ^a,b,c^, Qianqian Ouyang ^a,b,c^, Luming Deng ^a,b,c^, Hui Ma ^a,b,c^, Yuzhen Zhu ^a,b,c^, Fang Fang ^a^, Hua Ye ^a,b,c*^, and Kefeng Wu ^a,b,c*^

^a^ The Second Affiliated Hospital of Guangdong Medical University, Guangdong Medical University, Zhanjiang 524003, PR China

^b^ The Marine Biomedical Research Institute of Guangdong Zhanjiang, School of Ocean and Tropical Medicine. Guangdong Medical University, Zhanjiang 524023, China

^c^ Guangdong (Zhanjiang) Provincial Laboratory of Southern Marine Science and Engineering, Zhanjiang 524023, PR China

**Corresponding Author E-mail**:

[zhaoqi17@mails.ucas.ac.cn](mailto:zhaoqi17@mails.ucas.ac.cn); 27759157@qq.com; [winokhere@sina.com](mailto:winokhere@sina.com)


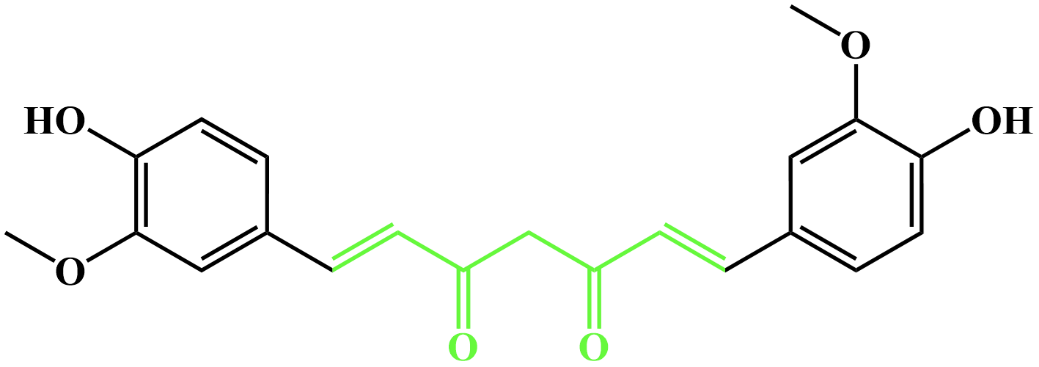


**Figure S1**. The chemical structure of CUR.


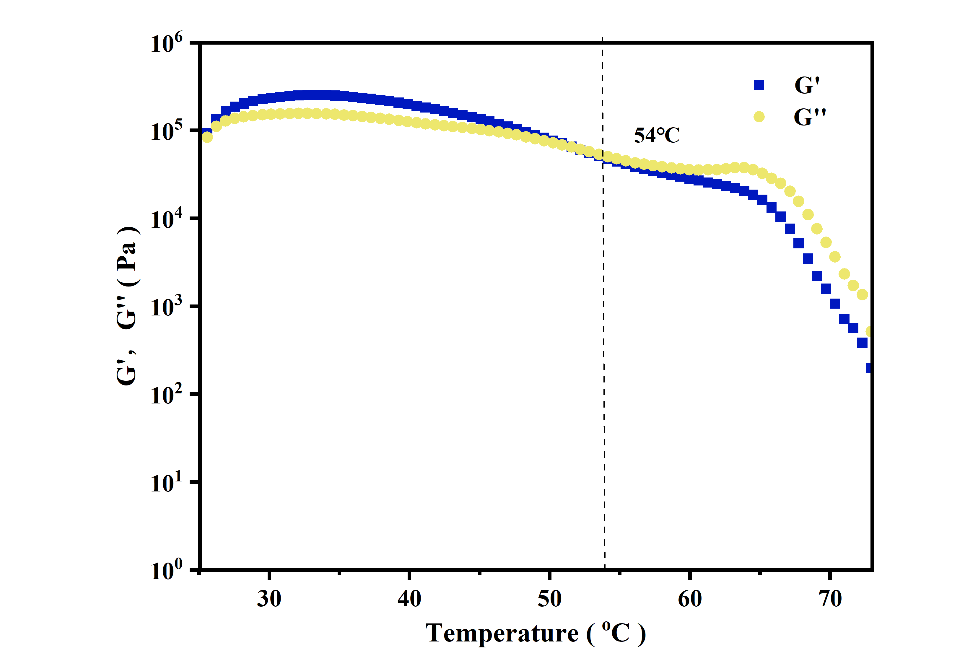


**Figure S2**. The rheological characterization of recovered gel from dried CUR-MS/DES gel.


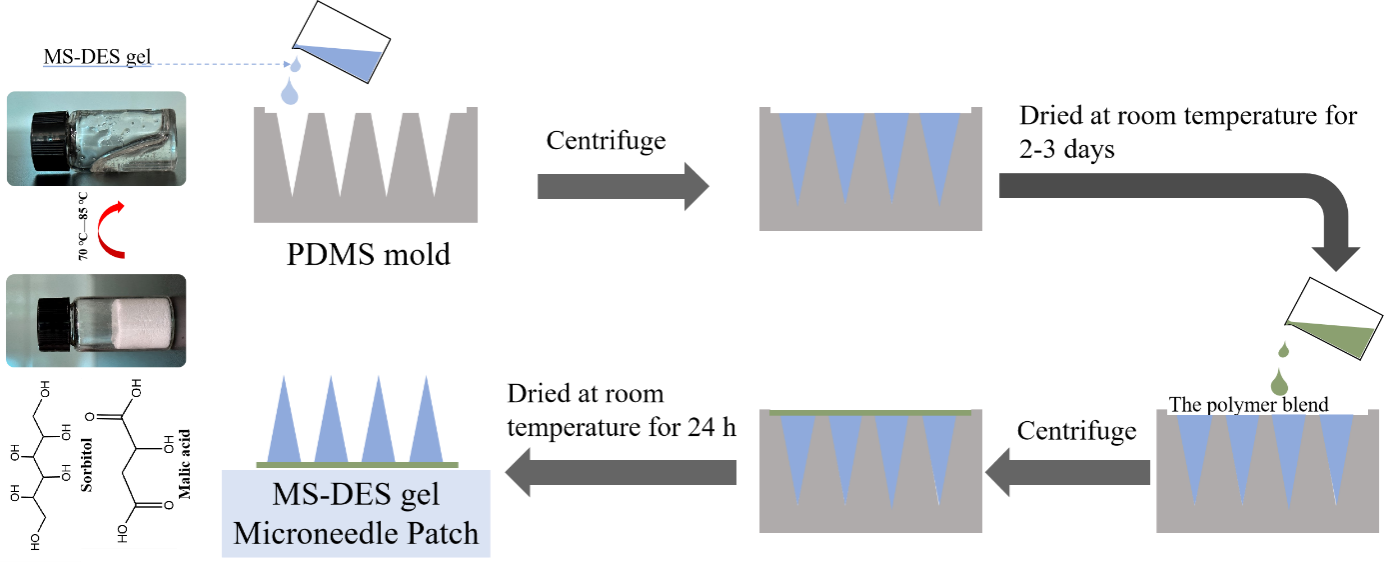


**Figure S3**. Schematic illustration of the fabrication process of the MS/DES-GMP.


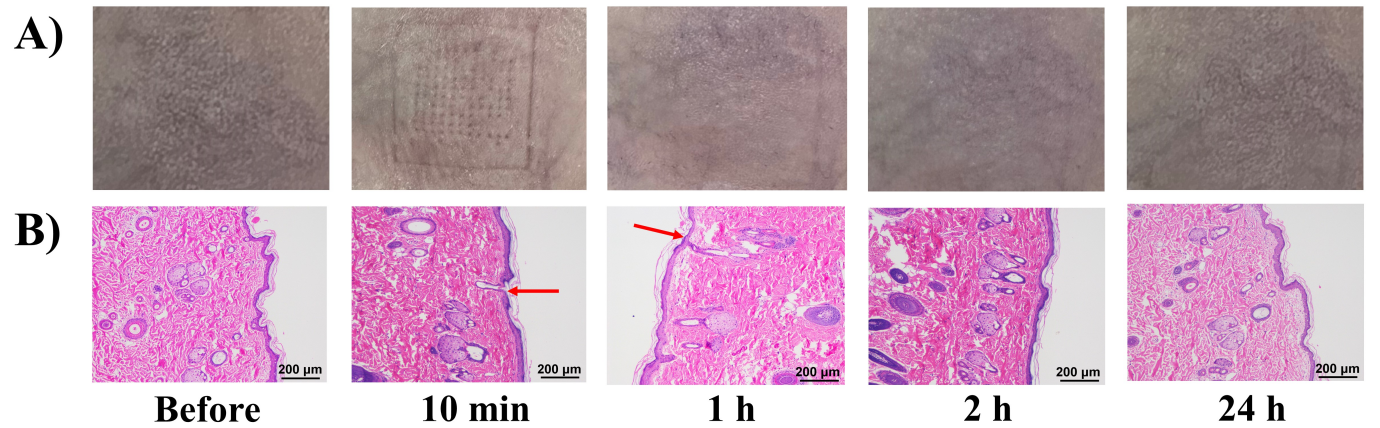


**Figure S4**. The continuous changes of CUR-MS/DES-GMN under the mice skin and the degree of wound healing during 24 h.
